# Ethical Considerations in the Management of Orthopedic Surgery Waste: Balancing Environmental Protection and Participant Safety

**DOI:** 10.7759/cureus.70342

**Published:** 2024-09-27

**Authors:** Ankita Kar, Apourv Pant, Rahul Shah

**Affiliations:** 1 Oral Medicine and Radiology, Indian Council of Medical Research, Assam, IND; 2 Bioethics, Indian Council of Medical Research, Bengaluru, IND; 3 Health Technology Assessment, Indian Council of Medical Research, Bengaluru, IND; 4 Orthopaedics, Grange University Hospital, Newport, GBR

**Keywords:** ethical considerations, orthopedic surgery, recycling and reuse in medical materials, sustainable healthcare practices, waste management

## Abstract

The healthcare sector significantly contributes to global greenhouse gas (GHG) emissions, with orthopedic surgery generating substantial waste, including single-use devices and hazardous materials. These practices exacerbate climate change and environmental degradation. This article explores the environmental and ethical implications of waste management in orthopedic surgery, focusing on the need for sustainable practices. Key approaches include recycling and reusing materials, implementing closed-loop supply chains, and promoting sustainable procurement. Case studies from various countries, such as Asia (India, Pakistan, Bangladesh), Africa, and Latin America, highlight the challenges and progress in managing medical waste, emphasizing the substantial potential for recycling preoperative waste. Ethical considerations revolve around ensuring safe waste handling to protect healthcare workers, patients, and communities while maintaining high standards of care. This should be done as per infection control and biomedical waste protocols.

National and international ethical guidelines extend their discussion to the principles of biomedical and health ethics (autonomy, beneficence, non-maleficence, and justice), especially regarding informed consent and the reuse of medical devices. Patients should be fully informed about environmental and waste management practices, with an emphasis on transparency and voluntary participation. The reuse of external fixators, while cost-effective, raises questions about ownership, safety, and cost transparency.

The article underscores the importance of adopting eco-friendly practices and green technologies in healthcare to mitigate the sector's carbon footprint. Initiatives such as energy-efficient buildings, renewable energy sources, and comprehensive recycling programs are vital. The conclusion calls for regulatory bodies and healthcare organizations to enforce guidelines for ethical waste management, balancing cost-effective practices with patient autonomy and environmental responsibility. Ethical waste management in orthopedic surgery is crucial for protecting the environment and the service providers and ensuring the well-being of all stakeholders involved.

## Editorial

The healthcare sector plays a significant role in contributing to the world's carbon footprint. It contributes more than 4.4% of net global greenhouse gas (GHG) emissions, impacting human health and the environment. Healthcare activities generate carbon dioxide (CO_2_) and other GHGs, which are collectively known as carbon dioxide equivalents (CO_2_e). These gases have the ability to trap heat within the atmosphere, and when present in excessive concentrations, they contribute to the negative effects of climate change. Orthopedic surgery generates a significant amount of waste, including single-use devices, sterilized materials, and hazardous substances. These materials can have severe environmental consequences when not managed properly. The extraction and production of medical materials, along with the disposal of waste, contribute to GHG emissions, resource depletion, and landfills overflowing with non-biodegradable medical waste. Single-use devices and disposable materials are convenient for healthcare providers, but they often consume more resources in their production and disposal than reusable alternatives. One key approach to reducing the environmental impact of orthopedic surgery is promoting the recycling and reuse of materials whenever possible. This includes reprocessing single-use devices, recycling plastics, and implementing closed-loop supply chains, etc., in medical equipment manufacturing. However, it raises ethical concerns regarding the ecological footprint of healthcare. Ethical considerations revolve around ensuring that waste is handled safely as per Guidelines for Management of Healthcare Waste, Biomedical Waste Management Rules, 2018-2023 to protect the well-being of healthcare workers, patients, and the community at large [1].

In this article, we will explore the ethical issues and considerations associated with waste management in orthopedic surgery, emphasizing the importance of both environmental protection and the protection of participants along with service providers.

Waste generation in the healthcare sector and its environmental implications

The volume of waste produced by hospitals varies significantly across countries. For instance, public hospitals in Greece generated 10,000 tons of hazardous waste in 2020, a figure projected to double by 2030. In a Brazilian university hospital, each bed generates 4.09 kg of waste daily, comprising 55.6% general waste, 39.1% infectious waste, 2.9% sharp waste, and 2.4% chemical waste [1].

In India, the healthcare sector is similarly challenged with waste management issues. Indian hospitals generate a significant amount of biomedical waste, with estimates suggesting that the country produces around 550 tons of medical waste daily. Of this, approximately 15% is categorized as hazardous. The management of this waste is crucial, as improper handling can lead to serious environmental and health hazards. Efforts to implement sustainable practices are ongoing, but challenges remain, including inadequate infrastructure and insufficient training for healthcare workers. Approximately 15% of healthcare waste is considered hazardous, posing risks if not managed properly. This waste can be infectious, toxic, or radioactive and can spread serious illnesses if not adequately treated [2].

The majority of the waste generated by the healthcare sector is also responsible for changing the global climatic regime as per 4.6% of total GHG emissions, which include CO_^2^_, methane, and ozone, among others. In the United States, where the share is 8.5%, the healthcare system is becoming more, not less, polluting: emissions increased 6% from 2010 to 2018 [3,4]. In the UK, the National Health Service (NHS) generates approximately 4% to 5% of the nation's total GHG emissions, which amounts to an estimated 25 megatonnes of CO_2_e annually. Furthermore, the production and disposal of medical waste also contribute to the sector's carbon footprint. The environmental impact of orthopedic surgery was studied in three areas: waste management, resource consumption, and carbon emissions [4].

The two most common barriers to implementing environmentally sustainable changes identified in the studies are the lack of appropriate infrastructure to support sustainable changes and the lack of education and training regarding sustainable practices.

The GHG Protocol offers another way to look at GHG emissions, categorizing them as follows: Scope 1: healthcare sector point and non-point emissions sources such as integrated biomedical waste treatment facilities and open landfill burning and dumping or accidental emission of medical waste. These emissions account for nearly 7% of overall health sector emissions; Scope 2: about 11% of indirect emissions from health care are generated by operational units that consume energy continuously to maintain health systems; Scope 3: indirect emissions were mainly caused by the manufacturing and mass production of medical supplies and their procurement through major pharmaceutical companies and industries, accounting for over 80% of the overall healthcare emissions [5].

Environment sustainability and waste management

The three Rs: reduce, recycle, and reuse

Literature states that there is a substantial potential for recycling preoperative waste generated by all orthopedic subspecialties. On average, 74% of the waste produced in the preoperative period was found to be recyclable. Operating rooms produce up to half of all hospital waste, with orthopedic surgeries being significant contributors. For example, knee arthroplasties in the United Kingdom generate over 2.7 million kg of waste annually, with only a small percentage recycled. The total life cycle carbon footprint of a knee arthroplasty is estimated at 190.5 kg of CO_2_. Effective recycling programs can reduce the ecological footprint of these procedures, demonstrating the importance of incorporating sustainability into healthcare practices. Kooner et al. (2020) stated that the waste material released after orthopedic surgery includes recyclable, non-recyclable, linen, and biological waste. The study also found that arthroplasty surgery produced substantially more biological waste per case than all other subspecialties. Phoon et al. (2023), in their scoping review, stated that studies have reported different types of waste streams, ranging from two to eight streams. In total, 1,824.7 kg of waste was generated. Recycled waste was also reported totaling 196.292 kg, out of which studies reported generating between 5.2 kg and 11.7 kg of sterile wrap, with only a few mentioning recycling [6,7].

Sustainable procurement

Sustainable procurement involves evaluating the lifecycle impacts of products from production to disposal, engaging with suppliers to ensure adherence to sustainable practices, prioritizing reusable and recyclable products to reduce waste, opting for energy-efficient products to cut costs and carbon footprints, and ensuring ethical labor practices. Implementation strategies include developing clear policies and guidelines, providing training and awareness for procurement staff, establishing performance metrics to measure success, collaborating with other institutions to share best practices, and continuously updating procurement practices to incorporate new sustainability standards and technologies.

Case Study: Sustainable Procurement in Indian Hospitals

In India, some hospitals have started to adopt sustainable procurement practices to address the challenges of resource consumption and waste management. For instance, a hospital in Delhi has implemented a policy to purchase energy-efficient medical equipment and use biodegradable materials in their canteen. These efforts have resulted in a 15% reduction in energy consumption and a significant decrease in non-biodegradable waste [8].

Additionally, several hospitals have begun sourcing locally produced medical supplies to support local economies and reduce the carbon footprint associated with transportation. These initiatives not only enhance the hospital's sustainability credentials but also foster positive relationships with local communities.

Healthcare institutions are increasingly prioritizing the procurement of products with minimal environmental impact, such as energy-efficient medical equipment, eco-friendly cleaning supplies, and sustainable packaging materials. By adopting sustainable procurement practices, healthcare facilities contribute to reducing their carbon footprint and supporting the growth of sustainable industries.

Mitigating the healthcare sector’s carbon footprint through ethical orthopedic practices

Addressing the carbon footprint of the healthcare sector is crucial for mitigating the negative impacts of climate change. Implementing sustainable practices and adopting green technologies can significantly reduce the sector's emissions. Several initiatives have already been undertaken to promote sustainability in healthcare, such as energy-efficient buildings, renewable energy sources, waste reduction and recycling programs, and the use of environmentally friendly materials.

One of the key initiatives promoting environmental sustainability in healthcare is the adoption of green building certifications. These certifications, such as Leadership in Energy and Environmental Design (LEED) and Building Research Establishment Environmental Assessment Method (BREEAM), ensure that healthcare facilities are designed, constructed, and operated using sustainable practices. Energy-efficient buildings such as improved insulation, energy-efficient lighting systems, and smart heating, ventilation, and air conditioning (HVAC) technologies [9].

Ethical considerations

The four principles which are respect for autonomy, beneficence, no maleficence, and justice form the ethical foundation of clinical ethics. Adhering to these principles ensures that any procedure is conducted with integrity, respect, and consideration for the well-being of patients. In the majority of countries, orthopedic surgeons have a legal obligation to obtain informed consent from patients before performing surgery. The Indian Council of Medical Research's (ICMR), National Ethical Guidelines, 2017, Declaration of Helsinki 2013 state that informed consent is a continuous process involving three main components: providing relevant information to potential participants, ensuring the competence of the individual, ensuring the information is easily comprehended by the participants, and assuring voluntariness of participation. Informed voluntary consent protects the individual’s freedom of choice and respects the individual’s autonomy [10].

The literature states that the ethical implications of reusing external fixation systems and other reusable equipment include considerations related to ownership of the implant, disclosure of reprocessed parts, infection risks, cost savings distribution, and humanitarian concerns. This raises critical questions about whether patients own their implants or if hospitals retain ownership for multiple uses, leading to substantial shifts in healthcare business relationships globally. 

Patients undergoing orthopedic surgery should be fully apprised of the environmental and waste management practices of the healthcare facility. Informed consent becomes pivotal, necessitating disclosure about potential reuse along with considerations for compensation, whether in cash or kind (ancillary care, free check-ups, or follow-ups). The reuse of external fixators can decrease costs, but questions arise about who owns the implant, who benefits from the savings, and the need to disclose the use of reprocessed parts.

There are also perceived concerns over safety and a lack of clarity in terms of cost transparency, making it challenging to have an informed opinion about the relative risks and benefits of single-use versus reprocessed external fixators. Patients should have the option to agree, disagree, or withdraw consent for the reuse of medical implants by hospitals/institutions, and they must be provided with a clear understanding of the associated benefits and risks. Vulnerable patients, who may not fully comprehend the complexities of the information presented in consent forms, may require a legally advised or authorized representative (LAR) to sign on their behalf. In cases of illiteracy, an impartial literate witness may be involved, and patients should not be subject to undue inducements.

Addressing ethical concerns, the reuse of external fixators can play a crucial role in providing access to implants in developing nations where such resources are scarce and traumatic injuries are prevalent. However, the ethical considerations surrounding safety, cost transparency, and the relative risks and benefits of single-use versus reprocessed external fixators need careful evaluation. Various research papers discuss the ethical considerations of reusing medical devices, including concerns about patient safety, regulatory compliance, informed consent, and financial considerations. Another study examines the ethical and legal considerations of reprocessing single-use medical devices, including the impact on patient safety, the necessity of informed consent, and the obligations of healthcare professionals [11].

Conclusion

The global initiatives undertaken to promote environmental sustainability in healthcare demonstrate the growing recognition of the industry's responsibility to protect the environment. Regulatory bodies and healthcare organizations should establish and enforce guidelines for waste management in orthopedic surgery. Transparent reporting and accountability mechanisms ensure that ethical standards are met. In conclusion, the ethical dimensions of orthopedic surgery extend beyond medical considerations to encompass environmental, economic, and humanitarian aspects. Achieving a balance between cost-effective practices and patient autonomy requires transparent communication, informed consent, and a commitment to ethical principles in the rapidly evolving landscape of healthcare.

Healthcare institutions and professionals must be proactive in adopting eco-friendly practices, recycling, and reducing waste, all while maintaining a high standard of care for patients. Ethical waste management in orthopedic surgery is not just about protecting the environment; it is also about safeguarding the well-being of all stakeholders involved in the process.
